# Expression of Senescence and Apoptosis Biomarkers in Synchronous Bilateral Breast Cancer: A Case Report

**DOI:** 10.3390/curroncol28050327

**Published:** 2021-09-30

**Authors:** Tareq Saleh, Mohammed El-Sadoni, Ahmad Alhesa, Heyam Awad, Mahmoud Jaradat, Mohammad Al-Hazaimeh, Rand Dawoud, Amel Mryyian, Bilal Azab

**Affiliations:** 1 Department of Basic Medical Sciences, Faculty of Medicine, The Hashemite University, Zarqa 13133, Jordan; 1636586@std.hu.edu.jo (R.D.); 1635542@std.hu.edu.jo (A.M.); 2 Department of Pathology, Microbiology and Forensic Medicine, School of Medicine, The University of Jordan, Amman 11942, Jordan; mhm8191130@ju.edu.jo (M.E.-S.); ahm8171602@ju.edu.jo (A.A.); h_awad@ju.edu.jo (H.A.); ba2659@cumc.columbia.edu (B.A.); 3 Department of General Surgery, Jordanian Royal Medical Services, Amman 11942, Jordan; mahmodjaradat@gmail.com (M.J.); dr.hazaimeh88.mh@gmail.com (M.A.-H.); 4 Department of Pathology and Cell Biology, Columbia University Irving Medical Center, New York, NY 10032, USA

**Keywords:** senescence, apoptosis, BCL-2, p16^INK4a^, chemotherapy, synchronous, bilateral, breast cancer

## Abstract

Background: Synchronous bilateral breast cancer (SBBC) provides a special condition where two independent breast tumors are exposed to cancer pharmacotherapy within a uniform pharmacokinetic milieu. Both senescence and apoptosis are established responses to therapy; however, they have potentially variable contributions to the overall outcome of treatment, which are yet to be determined. Methods: In this report, we describe the clinicopathological picture of two SBBC cases that received standard anticancer treatment and assess their expression profile of several molecular hallmarks of senescence and apoptosis. Results: Our analysis identified that synchronous tumors have variable expression profiles of both senescence- and apoptosis-associated biomarkers, despite comparable pathological responses to neoadjuvant chemotherapy and current survival rates. Conclusions: Our results highlight the variable expression of senescence- and apoptosis-associated markers in breast tumors (despite the shared somatic genetic background) and invites a large-scale assessment of both senescence and apoptosis in breast cancer tissue in vivo and their contribution to the pathological response and overall survival.

## 1. Introduction

Synchronous bilateral breast cancer (SBBC) is defined as the diagnosis of two independent primary breast cancer lesions (rather than contralateral metastasis) in both breasts, within an interval of one month to one year [1,2]. Despite the low frequency of SBBC (0.2–3% of all breast cancers), the incidence of SBBC has been rising due to improved diagnostic approaches [3,4]. The key risk factors of SBBC include younger age at presentation [5], a family history of bilateral breast cancer [6], lobular histology [7], multi-centricity [8], existence of sclerotic disease [4] and BRCA mutations [9]. Furthermore, an estrogen receptor (ER)-positive and human epidermal growth factor receptor 2 (HER2)- negative status was reported in the majority of SBBC tumors [10]. The prognosis of SBBC is largely undetermined, as some evidence supports a worse prognosis [11], while others suggest that the existence of a synchronous tumor only minimally affects overall survival [12]. Nonetheless, the five-year survival rate for synchronous breast cancer was around 60%, as opposed to 78.7% in metachronous bilateral breast cancer [5].

While the treatment of patients with SBBC is challenging due to the lack of established guidelines specific to the treatment of SBBC [13], many patients receive conventional neoadjuvant chemotherapy (NAC) as part of their therapy. More importantly, SBBC provides an avenue whereby two independent tumors, exposed to the same pharmacological cancer therapies, exist within the same pharmacokinetic environment. This allows for the investigation of several cell stress mechanisms that govern the level of pathological responses to NAC, especially given that a considerable percentage of patients receiving NAC fail to develop a complete pathological response (pCR) [14].

In this work, we report two cases of SBBC, of distinct tumor biology, with variable pathological responses to NAC, and utilize their tumor samples to investigate several biomarkers characteristic of two fundamental cell stress mechanisms: senescence and apoptosis. Apoptosis is the primary form of programmed cell death that mediates the cytotoxic effects of NAC [15,16] and accounts for the ideal development of pCR following pharmacotherapy. On the other hand, senescence has been increasingly recognized as a fundamental response to anti-cancer therapies [17], and a potential contributor to unfavorable therapy outcomes [18]. Interestingly, senescent cells are persistent, and resist apoptosis via upregulating the pro-survival proteins (e.g., BCL-2, BCL-X_L_) [19], which, consequently, renders them sensitive to senolytic agents that target these anti-apoptotic molecules [20]. Therefore, the interplay between senescence and apoptosis can partly explain the pathological outcome of a breast tumor following exposure to NAC [21]. Here, we provide evidence that synchronous breast cancer can have variable expression profiles of both senescence- and apoptosis-associated hallmarks, despite similar initial pathological responses to NAC. This report is the first to provide a comprehensive assessment of these biomarkers in SBBC cases. 

## 2. Case Presentation

The first case was of a 46-year-old premenopausal woman who presented to the breast surgery clinic at King Hussain Medical Center with bilateral breast masses with left-sided skin involvement and left axillary lymph node enlargement (Case 1, Table 1). Her mammography and ultrasonography revealed a dense mass of 3.1 × 2.4 cm in size, with irregular margins in the middle outer aspect of the left breast, enlarged pathological left axillary lymph nodes, and a lobulated mass of 2.4 × 1.7 cm in size along the upper outer quadrant of the right breast, which were classified BI-RADS 4 and 5 for the right and left breast masses, respectively. The core-needle biopsy of both breast masses established the diagnosis of invasive ductal carcinoma of the breast (IDC). Moreover, the immunohistochemical analysis of the hormone receptor expression status revealed that the right-sided breast tumor was positive for ER+, progesterone receptor (PR+) (100% and 30% expression level, respectively), and positive for HER2+. In contrast, the left-sided tumor was positive for both ER+ and PR+ (100% expression level for both), while negative for HER2-. Furthermore, fine-needle aspiration of the left axilla demonstrated the existence of malignant cells suggestive of a locally advanced breast carcinoma. However, staging studies were negative for evidence of distant metastatic disease. Thus, based on the 8th edition of the American Joint Committee on Cancer (AJCC) staging manual, the clinical staging was established as follows: stage IIIB (T4bN1M0) for the left-sided breast tumor and IIA (T2N0M0) for the right-sided breast tumor.

The second case was a 54-year-old postmenopausal woman who also presented to the breast clinic at King Hussain Medical Center with bilateral breast masses, associated with left nipple retraction, peau d’orange skin, and palpable axillary lymph nodes (Case 2, Table 1). Her mammography and ultrasonography revealed an irregular mass of 5 × 3.7 cm in size containing microcalcifications, resulting in tissue distortion and skin thickening in the upper outer quadrant of the left breast. Moreover, ultrasonography showed enlarged suspicious lymph nodes in the left axilla, and multiple ill-defined densities (the largest was 2.5 × 1.5 cm) with tiny calcifications in the lower inner quadrant of the right breast, categorized as BIRADS 5 in both breasts. The core-needle biopsy confirmed the diagnosis of bilateral IDC, whereas nodal metastasis was detected by FNA of the left axilla. The receptor status of the right-sided tumor was ER+, PR+ (100% for both), and HER2+. In comparison, the left-sided tumor exhibited lower expression of ER+ and PR+ (50% and 30%, respectively), and was negative for HER2-. No evidence of distant metastasis was detected. Accordingly, clinical staging based on the 8th AJCC staging manual was established as follows: IIIA (T2N2M0) for the left-sided breast tumor and IIA (T2N0M0) for the right-sided breast tumor.

Following the discussion of the treatment strategy by the onco-surgical multidisciplinary team, both patients received four cycles of NAC, namely, adriamycin + cyclophosphamide followed by docetaxel, trastuzumab, and pertuzumab. Two months following the completion of pharmacotherapy, Case 1 underwent a bilateral modified radical mastectomy (MRM) and bilateral axillary lymph node dissection. The histopathology of the right-sided tumor showed a residual invasive tumor of 17 mm in size, grade 2, 17 axillary lymph nodes negative for metastasis, no lympho-vascular or perineural invasion, and the receptor status was ER+, PR+, and HER2+ (ypT1c ypN0) (Table 1). The left side showed a tiny residual focus of an invasive tumor 2 mm in size, grade 2, two lymph nodes positive for metastasis out of 13, positive lympho-vascular and not identified perineural invasion, and the receptor status was ER+ and PR+ and HER2- (ypT1a ypN1a). On the other hand, Case 2 underwent bilateral MRM with axillary lymph node dissection, three months following the completion of pharmacotherapy. The surgical pathology report indicated the following: (1) right breast: residual tumor of 3 mm in size, grade 1, 22 axillary lymph nodes were negative for metastasis, no lymphovascular or perineural invasion, and the receptor status was ER+, PR+ (100% for both), and HER 2+ (ypT1a YpN0) (Table 1); (2) left breast: residual tumor of 7 mm in size, grade 3, 7 lymph nodes were positive for metastasis out of 13, lymphovascular and perineural invasion was detected, and the receptor status was ER+ and PR+ (50% and 30%, respectively), but was negative for HER2- (ypT1b YpN2a).

Based on the receptor status, both patients were commenced on postoperative trastuzumab and tamoxifen to decrease the risk of recurrence. Moreover, within three months of surgical intervention, both patients also received adjuvant radiotherapy with 50 Gy over 25 fractions (Table 1). To date (16 months and 11 months after treatment initiation for Case 1 and Case 2, respectively), both patients have remained asymptomatic, without clinical or radiological evidence of breast cancer recurrence. 

## 3. Biochemical Analysis 

**Figure 1:**
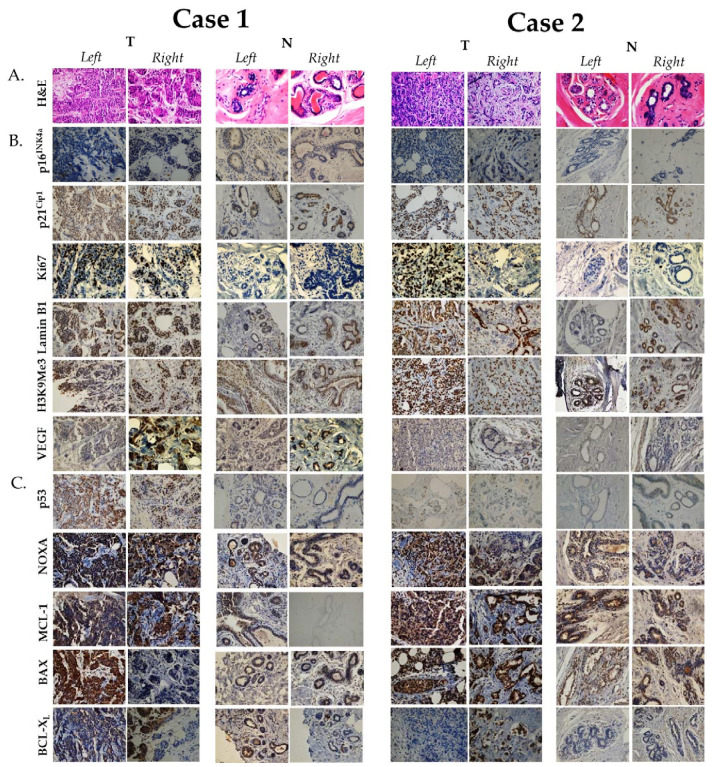
Immunohistochemical expression of senescence- and apoptosis-associated protein biomarkers in the two reported cases. (**A**). Hematoxylin and eosin (H&E) staining. (**B**). Senescence-associated biomarkers. (**C**). Apoptosis-related biomarkers. Immunohistochemistry was performed on true-cut (core-needle) formalin-fixed paraffin-embedded breast cancer biopsies of both cases prior to receiving NAC and as previously described [22]. Antibodies in this study were used at the following dilutions: anti-Ki67 (ab279653, 1:600dilution), anti-Lamin B1 (NBP2-59783, 1:250 dilution), anti-H3K9Me3 (6F12-H4, 1:200 dilution), anti-p21^Cip1^ (WA-1 (HJ21), 1:50 dilution), anti-p16^INK4a^ (1D7D2A1, 1:400 dilution), anti-VEGF (NB100-664, 1-30 dilution), anti-p53 (PAb 240, 1:300 dilution), anti-NOXA (114C307.1, 1:250 dilution), anti-BAX (NB100-56095, 1:1000 dilution), anti-MCL-1 (NB100-56146, 1:1000 dilution) and anti-BCL-X_L_ (NB-100-56104, 1:1000 dilution). All antibodies were obtained from Novus Biologicals, CO, USA, with the exception of anti-Ki67 antibody which was obtained from Abcam, MA, USA. Images were generated using a light microscope (Olympus BX 25, Olympus, Tokyo, Japan) under 40× objective magnification. Abbreviations: T: protein expression level in tumor (malignant) tissue; N: protein expression level in normal (non-malignant) breast tissue.

We then wanted to look at the expression level of several biomarkers associated with both senescence and apoptosis, aiming to identify variabilities in their expression level in SBBC, and whether it is linked to tumor response to therapy. For that, we employed immunohistochemistry techniques to detect and quantify the expression of these protein markers using true-cut and core-needle biopsy tumor (T) blocks for both cases, as described previously [22] (Figure 1). For comparison, we also provided an assessment of protein expression levels in normal breast epithelium (N) for each corresponding breast of both cases (Figure 1). The examined senescence-associated markers included: p16^INK4a^, p21^Cip1^, Ki67, Lamin B1, histone 3 lysine 9 tri-methylated (H3K9Me3) and vascular endothelial growth factor (VEGF) [23], while the examined apoptosis markers were: p53, NOXA, MCL-1, BAX, BCL-X_L_ [24].

For senescence, in Case 1, the right tumor was positive for p21^Cip1^ (T: 60%, N: 80%), Ki67 (T: 35%, N: negative), Lamin B1 (T: 60%, N: 80%), and H3K9Me3 (T: 60%, N: 90%) (Table 2). Similarly, the left tumor was positive for p21^Cip1^ (T: 5%, N: 50%), Ki67 (T: 65%, N: negative), Lamin B1 (T: 50%, N: 10%), and H3K9Me3 (T: <10%, N: 70%) (Table 2). The major differences in expression were in p21^Cip1^ (60% in the right tumor and 5% in the left tumor), VEGF (60% in the right tumor and negative in the left tumor) and H3K9Me3 (60% in the right tumor and <10% in the left tumor). Interestingly, both tumors were negative for p16^INK4a^ (Table 2). The markers that showed increased expression in malignant tissue as opposed to non-malignant include: Ki67 in both tumors, while the markers that exhibited reduced expression in malignant tissue as opposed to non-malignant include: p21^Cip1^ and H3K9Me3 (Table 2). Interestingly, Lamin B1 showed increased expression in the left tumor in contrast to its reduced expression in the right tumor relative to its expression in normal breast epithelium. In Case 2, the right tumor was positive for p21^Cip1^ (T: 90%, N: 60%), Ki67 (T: 5%, N: negative), Lamin B1 (T: 90%, N: 90%), and H3K9Me3 (T: 90%, N: 85%), while negative for p16^INK4a^ (Table 2). In comparison, the left tumor was positive for, p21^Cip1^ (T: 70%, N: 70%), Ki67 (T: 70%, N: negative), Lamin B1 (T: 90%, N: negative), and H3K9Me3 (T: 90%, N: 95%), while negative for VEGF and p16^INK4a^ (T: only 2% expression level, N: negative) (Table 2). The major difference in expression between the two cases was in Ki67 (5% in the right tumor and 70% in the left tumor), Lamin B1 (higher in both tumors of Case 2) and H3K9Me3 (higher in both tumors of Case 2). No remarkable differences in the expression level of proteins between malignant and non-malignant tissue was observed except for Ki67, which is expected to be elevated in tumor cells, and Lamin B1 in the left tumor.

For apoptosis, in Case 1, the right tumor was positive for p53 (T: 45%, N: <1%), NOXA (T: 40%, N: 5%), MCL-1 (T: 80%, N: negative), BAX (T: <10%, N: 10%) and BCL_-_X_L_ (T: 40%, N: <1%) (Table 2). Similarly, the left tumor was positive for p53 (T: 75%, N: negative), NOXA (T: 60%, N: 5%), MCL-1 (T: 50%, N: negative), BAX (T: 70%, N: negative) and BCL_-_X_L_ (T: 30%, N: 5%). The major differences in expression were in p53 (45% in the right tumor and 75% in the left tumor) and BAX (<10% in the right tumor and 70% in the left tumor). All markers exhibited increased expression in malignant tissue as opposed to non-malignant apart from BAX. In Case 2, the right tumor was positive for NOXA (T: 80%, N: negative), MCL-1 (T: 70%, N: 50%), BAX (T: >90%, N: 10%) and low for BCL_-_X_L_ (T: <5%, N: negative), while the left tumor was positive for NOXA (T: 90%, N: 1%), MCL-1 (T: 50%, N: 80%), BAX (T: >90%, N: 30%) and low for BCL_-_X_L_ (T: <5%, N: negative) (Table 2). No major differences between the expression profile of apoptosis markers between the two tumors of Case 2, except for MCL-1, the expression of which was decreased in the left tumor relative to non-malignant breast tissue. In addition, p53 was expressed in less than 1% of breast tumor cells. Accordingly, a major difference between the two cases was p53 expression (very low in both tumors of Case 2) and BCL_-_X_L_ (lower in both tumors of Case 2) (Table 2).

The table shows expression levels of the indicated biomakers based on the number of positive cells in the histological section, as semi-quantitatively assessed by two pathologists independently. p16^INK4a^ and p21^Cip1^ are cyclin-dependent kinase inhibitors (CDKIs) that regulate the senescent growth arrest [23]. Ki67 is a proliferation marker that is often expressed at low levels in senescent cells [23]. Lamin B1 is a component of nuclear lamina and is often degraded in senescent cells. Histone H3 tri-methylation at the 9th lysine (H3K9Me3) is an epigenetic modification that is part of the Senescence-Associated Heterochromatic Foci, while the vascular endothelial growth factor (VEGF) is a frequently secreted protein and is part of the Senescence-Associated Secretory Phenotype (SASP) [23]. p53 is a common regulator of both senescence and apoptosis. BCL_-_X_L_ is mitochondrial transmembrane anti-apoptotic (pro-survival) protein part of the BCL-2 family [15]. MCL-1 is an apoptosis regulatory protein that is implicated in mitochondrial homeostasis and part of the BCL-2 family. BAX is a pro-apoptotic protein and member of the BCL-2 family, responsible for increasing the permeability of the mitochondrial membrane and release of cytochrome c [15]. NOXA is a pro-apoptotic protein involved in p53-mediated apoptosis [15]. Abbreviations: T: protein expression level in tumor (malignant) tissue; N: protein expression level in normal (non-malignant) breast tissue; (−): negative expression.

## 4. Discussion

SBBC represents a unique condition where two independent tumors arise simultaneously in the same patient [1,2]. Despite its relatively improbable occurrence, SBBC provides an avenue to study treatment outcomes and associated cellular and molecular processes in a uniform pharmacokinetic and genetic environment. Furthermore, the prognosis of SBBC is still not well-defined, and thus, reporting the variability of tumor response to therapy and the consequent overall survival is of relevance. In this work, we report two cases of SBBC that received standard breast cancer treatment and responded favorably to therapy. The two cases were relatively young (Case 1 was 46 years old and Case 2 was 54 years old) which is consistent with SBBC being more common in younger females [5]. Moreover, both cases were hormone receptor positive (ER+, PR+), which is classical for SBBC [10], but had variable HER2 expression. Unfortunately, evidence for family history of bilateral breast cancer or BRCA status was not possible to determine for these patients, although SBBC is most likely to occur in patients with previous family history [6], or who have BRCA mutations [9].

Both cases received four cycles of NAC (Adriamycin + cyclophosphamide, followed by docetaxel), before undergoing bilateral MRM and axillary lymph nodes dissection followed by adjuvant radio and hormonal therapy, which is standard-of-care for breast cancer. Both cases developed tumor regression in response to NAC and none of the tumors developed pCR (Table 1). However, the overall response to therapy has been reassuring so far, where both patients have remained asymptomatic and without clinical or radiological evidence of breast cancer recurrence. This is in consistence with previous reports that showed the minimal effect on overall survival, which is attributed to the existence of a synchronous tumor [12]. However, due to the limited follow-up period, the five-year survival was not determined, and consequently, a worse overall prognosis cannot be ruled out [11].

Next, our analysis has identified variable expression profiles of senescence- and apoptosis-related biomarkers. For example, there was a high variability in the percentage of expression of p21^Cip1^ between the two tumors of Case 1 (Case 1/left: 5%, Case 2/right: 60%). This difference in expression was observed in SBBC previously, however, overall p21^Cip1^ expression levels were higher in SBBC in comparison with metachronous bilateral breast cancer [25]. Interestingly, p16^INK4a^ was negative across all tested tumor samples. p16^INK4a^ expression is known to be lost in multiple types of malignancies due to frame-shift mutations. When p16^INK4a^ is present nonetheless, it is likely to accumulate in breast tumor cells in response to NAC, indicative of senescence induction [26]. It is important to note that the presence of functional p16^INK4a^ is not an absolute pre-requisite for tumor cells to undergo senescence, as the growth arrest can be ensued through other pathways e.g., the p53/ p21^Cip1^ axis. Furthermore, p16^INK4a^ is a fundamental tumor suppressor gene and its loss of expression/function is believed to happen in the escape from oncogene-induced senescence and transformation, as in the case with p21^Cip1^ [27]. Finally, the expression of Lamin B1 was in agreement with our previous findings in IDC samples [22]. The accumulation of senescent cells as part of the tumor biology or as a byproduct of cancer chemotherapy, has been identified lately as an unfavorable mechanism that accounts for several adverse outcomes of cancer therapy, including the risk for recurrence [18]. Accordingly, the removal of senescent tumor cells was proposed as a novel therapeutic strategy using newly-identified senolytic drugs [28]. However, the proper, more individualized, implementation of senolytic therapy would require a readily identification of tumor cell senescence in vivo, especially given that data from this report, and our previous results [22], suggest that senescence is not a universal component of tumors, especially of the breast. Importantly, our results only represent an initial assessment of the expression of biomarkers related to both senescence and apoptosis, and thus, it is challenging to come to any conclusion on the basis of data from only two patients’. This study should only be viewed as preliminary and require further large-scale analysis to propose any conclusion for breast cancer markers.

Relevant to the incorporation of senolytics in cancer therapy, many of the most effective senolytics are BH3 mimetics, which interfere with apoptosis-regulating members of the BCL-2 protein family [21,24]. In fact, senescent cells show higher expression levels of anti-apoptotic protein such as BCL-2 and BCL-X_L_, which accounts, in part, for their apoptosis resistance and persistence in culture (possibly in vivo) for long periods [29]. Therefore, in this study, we wanted to examine several key components of the apoptosis regulatory pathway. Interestingly, BCL-X_L_ expression level was lower in both Case 2 tumors in comparison to Case 1 tumors. BCL-2 and BCL-X_L_ expressing tumor cells have definitely been identified as targets for senolytic agents, such as ABT-263 and ABT-737 [28,30]. Elevated MCL-1 expression level has been associated with poor outcomes in breast cancer patients. In this report, average MCL-1 expression level was identified in both cases [31]. Lafontaine et al. has suggested that the senolytic effect of BH3 mimetics might be dependent on functional MCL-1 [32]. Similarly, Shahbandi et al. demonstrated that low NOXA expression leads to resistance to BH3 mimetics, which would require further MCL1 inhibition to exert successful senolysis [33]. Our study shows a relatively lower level of NOXA in Case 2 tumors. Since both BCL-X_L_ and NOXA levels are low in Case 1, the expectation is that the patient will respond less to BCL-2-targeting senolytics. However, this is dependent on whether Therapy-Induced Senescence (TIS) was an outcome of NAC. In fact, an important limitation in our study is the inability to look at the senescence- and apoptosis-related markers in the postoperative tumor samples, due to the semi-complete response to NAC and lack of sufficient tumor tissue for biochemical analysis. 

## 5. Conclusions

SBBC can be a vehicle for the identification of differences in tumor biology. Both senescence and apoptosis are fundamental mechanisms that contribute to treatment outcomes of cancer. To our knowledge, this is the first report to provide a comprehensive assessment of these biomarkers in SBBC cases. We reported variability in the expression levels of protein markers associated with both phenotypes within bilateral tumors of the same patient, suggesting that the induction of these processes can be highly heterogenous. These results invite for large-scale studies that can identify holistic spectrums of apoptosis- and senescence-associated marker expression and establish a correlation between their induction and potential overall outcomes of cancer treatment. Lastly, the identified heterogeneity requires the development of readily available means to identify senescence in vivo and to utilize senolytic therapy in an individualized manner.

## Figures and Tables

**Table 1:** Comparison of different diagnostic and treatment characteristics between the two reported cases.

	Case 1		Case 2	
	Right	Left	Right	Left
BI-RADS	4	5	5	5
Diagnosis	IDC	IDC	IDC	IDC
Grade	G1	G3	G1	G3
TNM Staging	T2N0M0	T4bN1M0	T2N0M0	T2N2M0
Receptor Status	ER+, PR+, HER2+	ER+, PR+, HER2-	ER+, PR+, HER2+	ER+, PR+, HER2-
NAC Regimen	A+C (4 cycles) followed by D+T+ P		A+C (4 cycles) followed by D+T+ P	
Surgical Intervention	Bilateral MRM + axillary lymph nodes dissection		Bilateral MRM + axillary lymph nodes dissection	
ypTNM Staging	ypT1c ypN0	ypT1a ypN1a	ypT1a ypN0	pT1b ypN2a
Adjuvant therapy	Radiotherapy 50 Gy over 25 fractions trastuzumab and tamoxifen		Radiotherapy 50 Gy over 25 fractions trastuzumab and tamoxifen	

The table describes the clinicopathological characteristics of both tumors, neoadjuvant chemotherapy, adjuvant therapy regimens and mode of surgical intervention of both reported cases. The table also presents the intraoperative pathological staging following the completion of treatment with NAC. Patients’ information was collected from the databases of King Hussein Medical Center based on the approval by the Institutional Board Review (IRB) committee of the Jordanian Royal Medical Services (JRMS) No. 6/2021 and in accordance with the World Medical Association Declaration of Helsinki. Both participating subjects provided informed consent. Abbreviations: BI-RADS: Breast Imaging Reporting and Data System; IDC: invasive ductal carcinoma; G: grade; ER: estrogen receptor; PR: progesterone receptor; HER2: human epidermal growth receptor 2; A: Adriamycin; C: cyclophosphamide; D: docetaxel; T: trastuzumab; P: pertuzumab; MRM: modified radical mastectomy; Gy: gray.

**Table 2:** Expression levels of senescence- and apoptosis-associated protein biomarkers in the two reported cases.

	Case 1				Case 2			
	T		N		T		N	
	Left	Right	Left	Right	Left	Right	Left	Right
p16^INK4a^	−	−	−	1%	2%	−	−	−
p21^Cip1^	5%	60%	50%	80%	70%	90%	70%	60%
Ki67	65%	35%	−	−	70%	5%	−	−
Lamin B1	50%	60%	10%	80%	90%	90%	−	90%
H3K9Me3	<10%	60%	70%	90%	90%	90%	95%	85%
VEGF	−	60%	−	25%	−	−	−	−
p53	75%	45%	−	<1%	1%	1%	−	−
NOXA	60%	40%	5%	5%	90%	80%	1%	−
MCL-1	50%	80%	−	−	50%	70%	80%	50%
BAX	70%	<10%	−	10%	90%	90%	30%	10%
BCL-X_L_	30%	40%	5%	<1%	<5%	<5%	−	−

## Data Availability

The data presented in this study is available on request from the corresponding author. The data is not publicly available due to patient privacy.
